# Phenotyping antidepressant treatment response with deep learning in electronic health records

**DOI:** 10.1038/s41398-026-04266-1

**Published:** 2026-07-11

**Authors:** Yi-han Sheu, Colin Magdamo, Matthew Miller, Sudeshna Das, Deborah Blacker, Jordan W. Smoller

**Affiliations:** 1 Center for Precision Psychiatry, Massachusetts General Hospital, Boston, MA USA; 2 Psychiatric and Neurodevelopmental Genetics Unit, Center for Genomic Medicine, Massachusetts General Hospital, Boston, MA USA; 3 Department of Psychiatry, Massachusetts General Hospital/Harvard Medical School, Boston, MA USA; 4 Stanley Center for Psychiatric Research, Broad Institute of MIT and Harvard, Cambridge, MA USA; 5 Department of Neurology, Massachusetts General Hospital/Harvard Medical School, Boston, MA USA; 6 Bouvé College of Health Sciences, Northeastern University, Boston, MA USA; 7 Harvard Injury Control Research Centre, Harvard T.H. Chan School of Public Health, Boston, MA USA; 8 Department of Epidemiology, Harvard T.H. Chan School of Public Health, Boston, MA USA

**Keywords:** Depression, Diagnostic markers

## Abstract

Efficient, accurate phenotyping for antidepressant treatment response in electronic health records (EHRs) could facilitate precision psychiatry applications but remains a challenge. Increasingly, artificial intelligence methods using deep learning applied to clinical data have shown promise in complex classification problems. Here, we systematically evaluate the performance of eight deep learning-based language models in classifying response to antidepressants in a large real-world healthcare setting. We obtained data spanning 1990–2018 for adults with depression and a co-occurring antidepressant prescription from the EHR data warehouse of the Mass General Brigham healthcare system (n = 111,563). Clinical note sets (n = 3996) were collected for 4–12 weeks after antidepressant initiation, and were manually reviewed to classify response status as “improved” or “no evidence of improvement” in depression symptoms for model development and evaluation. The phenotyping models performed well, with the majority having areas under the receiver operator curve (AUROC) exceeding 0.80. For example, BERT-large with sliding window had an AUROC = 0.84 and PPV = 0.80 at a specificity of 0.87. Our results indicate that deep learning language models applied to EHR data can accurately classify antidepressant response in a real-world healthcare setting.

## Introduction

Depression is a prevalent psychiatric disorder associated with substantial disability and healthcare costs [1]. Real-world electronic health record (EHR) data has strong potential to address unmet needs in depression care, enabling applications such as response prediction models for treatment selection [2], emulated target trials to estimate comparative effectiveness [3, 4], and linkage to biobanks for large-scale genetic discovery [5, 6]. Nevertheless, these downstream uses require a prerequisite step: accurately identifying (“phenotyping”) antidepressant treatment response in routine-care data.

Manual extraction of response data from large health system cohorts is, however, prohibitively costly and difficult to scale, and making an effective method for automated classification of treatment could be highly beneficial. A major obstacle is that structured EHR data (e.g., medications, problem lists, ICD billing codes), unlike data collected specifically for research purposes such as randomized trials [7], do not readily provide information relevant to treatment response. For example, Perlis and colleagues (2012) found that billing codes performed only slightly better than chance at classifying antidepressant-related remission of depression (area under the receiver operating characteristic curve (AUROC) ~ 0.55) [8]. Other proxy approaches based on medication switching or augmentation in structured prescription records are conceptually limited because regimen changes may reflect factors other than response (e.g., adverse effects, adherence, access, or patient preference).

Because response is often documented in free-text clinical notes, a scalable natural language processing (NLP)-based phenotyping algorithm that extracts antidepressant response from EHR text can provide reliable outcome labels at scale impractical with manual review, unlocking critical downstream use cases. However, the optimal method for incorporating NLP in this setting remains unclear. In contrast to conditions that may be well captured specific medical terms, text related to depression and treatment response may involve highly heterogeneous, time-varying terms (including non-specific descriptions of symptoms). Further, while some response-related text could be encoded by short statements (e.g., “depression: doing well”), it may require extraction of information from longer narrative paragraphs, requiring subtle syntactical and semantical analysis. Classical NLP methods, which typically involve modeling with a “bag of words” (e.g., the presence or counts of words, without considering temporal order) fail to account for contextual information.

Recently, deep learning-based NLP models (or “language models”) have achieved paradigm-shifting results in general NLP tasks [9, 10]. These models use text information beyond seeking a fixed set of terms. Instead, each word is pre-injected with contextual and syntactical information, and the model automatically learns which are related to the prediction output. These methods can also accommodate lexical derivatives and typographical errors without the need for manual curation.

To assess whether deep learning-based NLP can classify antidepressant treatment response from unstructured clinical notes and thereby approximate chart-review, we evaluated Transformer-based models using narrative EHR documentation and quantified performance. Our primary hypothesis is that deep learning-based NLP models can effectively emulate an expert reviewer’s assessment of antidepressant treatment response. We also conducted a sensitivity analysis assessing model performance on patient distributions that differed from those used in training. Because the vast majority of antidepressant treatments are not initiated by psychiatrists, we focused on patients who started treatment in non-psychiatric settings as our intended use case [11]. We show that these models can achieve good discrimination performance for antidepressant response using clinical notes.

For the reader’s convenience, we provide a glossary of technical terms related to deep learning or defined specifically for this work in eTable 1.

## Methods

### Data source and cohort definition

The study data were extracted from unstructured (i.e., free text) clinical note data available in the Mass General Brigham (MGB) Research Patient Data Registry (RPDR) [12]. The RPDR is a centralized data warehouse that gathers clinical information from hospitals in the MGB system and currently includes more than 7 million patients with over 3 billion records seen across ten hospitals, including two major Harvard teaching hospitals, Massachusetts General Hospital and Brigham and Women’s Hospital.

Clinical notes were requested from the RPDR for patients aged 18 or older who had at least one visit with an International Classification of Diseases-Clinical Modification (ICD-CM) code for a depressive disorder (ICD-9-CM: 296.20-6, 296.30-6, and 311; ICD-10-CM: F32.0-9, F32.81, F32.89, F32.8, F33.0-3, and F33.8-9) and an antidepressant prescription recorded during the same encounter during 1990–2018, to increase the likelihood that the antidepressant was prescribed for depression rather than other indications. We additionally required at least one ICD code for non-recurrent depression (ICD-9-CM: 296.20–296.26 and 311; ICD-10-CM: F32.0–F32.9) at any time in the patient record to reduce the likelihood of including prevalent antidepressant users, consistent with our focus on incident treatment initiation. We limited the sample to adults because diagnostic coding practices, treatment indications, and documentation patterns differ substantially in pediatric populations. We excluded patients prescribed more than one antidepressant on the index date to reduce the likelihood of including prevalent users or complex treatment regimens that may indicate ongoing therapy prior to capture in RPDR, which could confound interpretation of early treatment response. Patients whose treatment was initiated in a psychiatric setting were excluded to focus on our primary use case.

The notes included office visits, admission notes, progress notes, discharge notes, and correspondence from all medical specialties in the MGB system. The clinical notes come in many varieties and can contain a wide range of information such as the patient’s chief complaint; history of present illness; the physician’s examination and observations; the physician’s assessment and treatment plans, which may or may not include a list of active problems; current and past medications with or without doses; and patient-reported outcome measures, in the form of questionnaires that were converted to text and merged with other sources of clinical notes.

### Data sampling and labeling for modeling classification of treatment response

For labeling and modeling of treatment response classification, we obtained prescription information from the RPDR and identified the first date of prescription (index date) of an antidepressant for each patient to anchor follow-up and to support a consistent operational definition across patients.

**Fig. 1:**
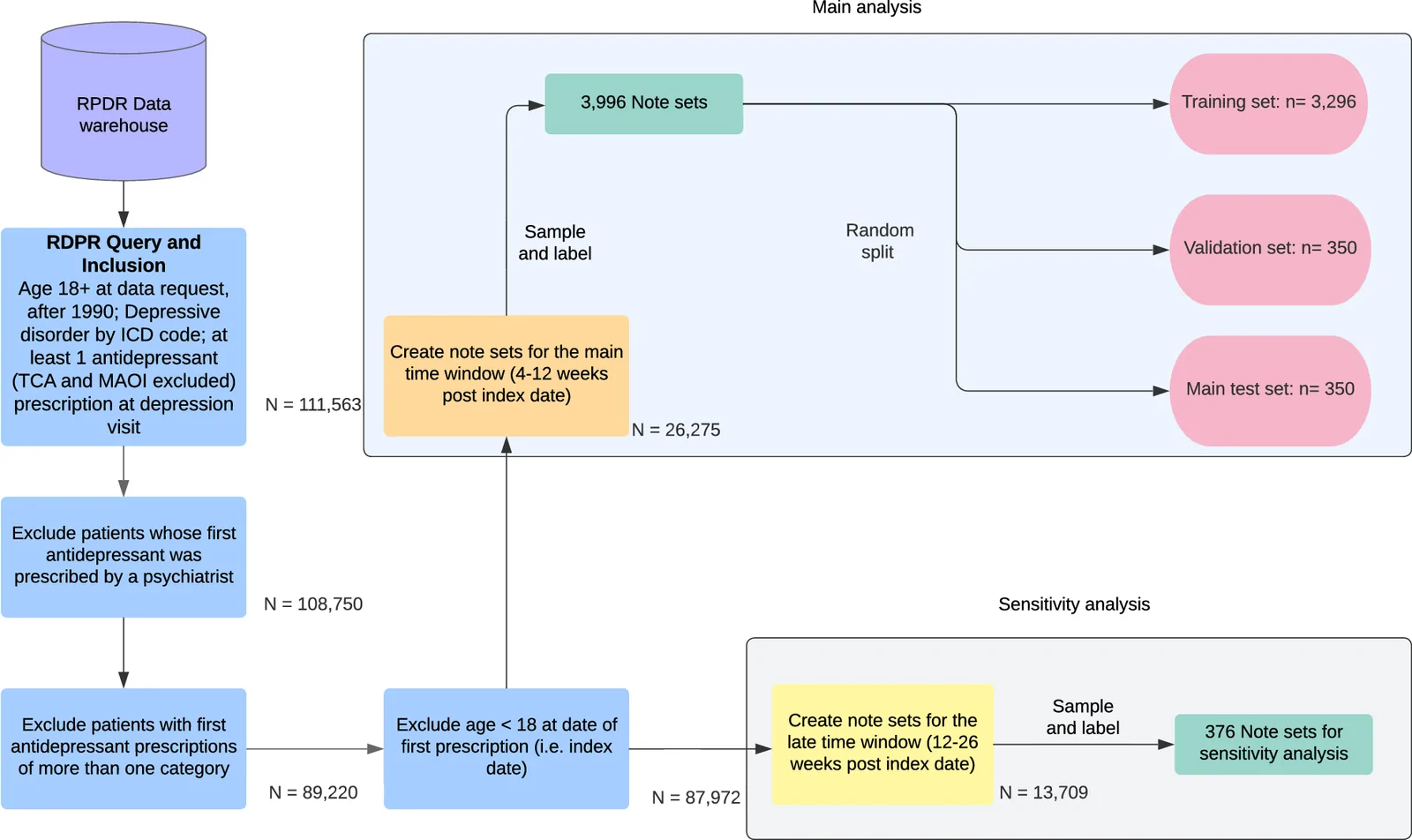
Flow diagram for data retrieval and sampling/construction process of the note sets.

Clinical notes collected from 4–12 weeks following the index date were concatenated into “note sets” for each patient. This period represents the standard timeframe for reliably assessing treatment response and guiding clinical decisions, and it is the primary focus of the analysis (referred to below as the “main time window”). Each note set exclusively contains notes from a single, unique patient (no additional structured data was included). Within this time window, we randomly sampled note sets from 3996 patients to obtain a labeled dataset broadly representative of the eligible population. An expert psychiatrist (YHS) manually reviewed and labeled the sampled note sets, as described below. The sample size was selected to balance the feasibility of manual chart review with the need for adequate statistical power to train and evaluate NLP models. Figure 1 provides a flow diagram for data retrieval and sampling/construction process of the note sets.

We defined two classes for our classification labels as follows: class (1), Improved: there was evidence in the note set that the patient responded to treatment based on an assessment of their current status, defined by descriptions of depressive symptoms changing in a positive way (e.g., “depression symptoms improved,” “depression well controlled,” or “on X medication and felt much better”); class (2), No evidence of improvement: there was either information in the note set describing mood status, but no evidence of a positive response (e.g., “patient continues to have low mood after starting X medication six weeks ago”) or apparent depression-related symptoms are recorded in the note set without any mention of change (e.g. “this visit, the patient came in stressed and crying, complaining of persisting sleep problems…”), or information is lacking or insufficient in the note set regarding mood status (e.g., there are visits for other conditions that do not provide any assessment of mood or mental status, or the patient’s condition does not allow proper assessment of mood [e.g., due to change in consciousness]), or in the rare case, if the patient was noted to have gotten worse. During labeling, if there were two or more mentions of depression status in the note set over time, the latest one with any description of mood state was chosen for labeling.

Note that because the note sets included clinical notes from all specialties during the specified time window (not restricted to mental health specialty care), information specifically related to depression typically constituted only a small proportion relative to the total text volume in each note set. This relative sparsity is addressed by the self-attention mechanism [13] inherent in the NLP models employed in our study (BERT [9], ClinicalBERT [14], and Longformer [15]; see below).

### Text preprocessing

In the clinical notes, all non-alphanumeric characters were removed, except for “.” And “-”, which were used by the models. In addition, identifying information (names, medical record numbers, providers, etc.) was removed.

Before the note sets were used as input to a deep learning model, they were first converted to “tokens,” which are short sequences of characters that may be a number, a word, a fragment of a word, or a punctuation symbol (see Glossary for further definition of deep learning terms). Each token was then converted to an “embedding”—that is, a numeric vector that carries prior information about the token [9]. A simple example of an embedding would be information on how often a given word (e.g. “apple”) co-occurs with other specific words (e.g. “fruit”, “banana”, etc.). Tokens that carry similar information are converted to vectors that are similar. The conversion of text to tokens was done by a “tokenizer.” [9] The specific choice of tokenizer corresponded to the downstream deep learning model applied (i.e., the BERT or Longformer tokenizers, see below). The tokens were converted into pre-trained embeddings using the built-in embedding mechanisms specific to BERT (WordPiece embeddings [9]) and Longformer (Byte-level Byte-Pair Encoding, BBPE [15]).

### Models for classification of treatment response

The model classes we utilized in this study are based on “Transformers.” [13] The Transformer is a model architecture that heavily utilizes the mechanism of self-attention [13]. Self-attention measures similarity between each token in the text, and tokens that are similar and relevant to the task are amplified to produce the final model prediction. This approach has proven to be highly effective for sequential data and is the state-of-the-art approach for current deep learning-based NLP models [9, 13].

**Fig. 2:**
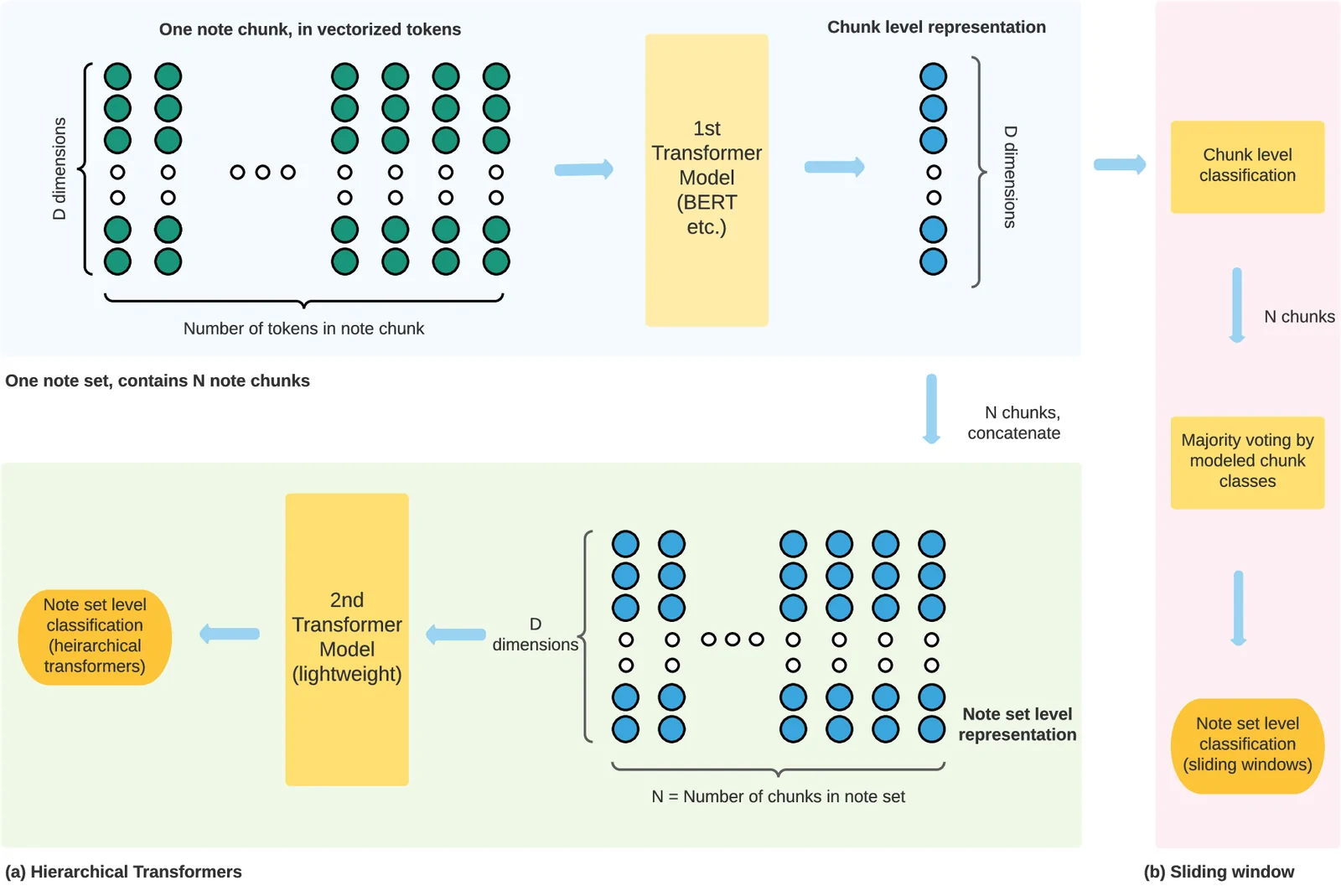
Illustration of information flow for two modeling strategies. Each note set was split into shorter “chunks” for processing. A first level Transformer model was used to derive chunk level representation and classification. **a** The hierarchical Transformers approach (green background) collects all chunk level representations and processes them through a second level lightweight Transformer model to derive note set level classification. **b** The sliding window approach (pink background) collects all chunk level classification labels for a given note set and performs majority voting to derive note set level classification (right hand side). D: number of dimensions used per token. BERT-base and ClinicalBERT: 768, Bert-large and Longformer-large: 1024. N: number of chunks in each note set (which can vary between different note sets).

However, due to their computational burden, Transformer models often pose upper limits on the sequence length (i.e., the number of tokens) they can intake at once, and the length of the note sets often exceeded these upper limits (eTable 2). To address this, we adopted the following two approaches (based on Pappagari et. al. [16]): (1) the “sliding window” approach, and (2) the “hierarchical Transformers” approach (Fig. 2). We trained and optimized models using the training and validation sets, and report test set model performance for four models—BERT-base [9], ClincalBERT [14], BERT-large [9], and Longformer-large [15]—under each of the two aforementioned approaches (a total of 8 models).

The BERT model is a large stack of Transformers intertwined with feedforward layers [17], which are the basic components of deep neural networks whereby all artificial neurons from the previous layer are connected to all of those in the next layer. BERT was pre-trained on large scale real world corpora (i.e., Wikipedia and BookCorpus [18, 19]), and therefore its model weights already carry semantic and syntactic information about these corpora before BERT is applied to any downstream task. It has demonstrated excellent performance in a variety of NLP tasks [9] and is still used as a common reference model for NLP tasks. BERT has two versions: BERT-base and BERT-large, where BERT-large utilizes a greater number of dimensions representing each token compared to BERT-base (1024 vs 768), allowing more information to be carried in each token. In addition, BERT-large has a larger number of Transformers stacked. ClinicalBERT (specifically, the “Bio+Clinical BERT” variant) is a modified version of BERT-base [14]: in addition to general text, it has been further pre-trained on PubMed abstracts, PubMED Central full texts, and publicly available clinical notes from an intensive care unit (ICU), MIMIC-III [20], and theoretically carries more healthcare-specific information inside its model weights. While the content and structure of the ICU-derived notes used to pre-train ClinicalBERT may differ from the broader EHR note set included in the RPDR, they contain specific clinical and medical details not typically found in general text corpora. Thus, we felt it worthwhile to evaluate ClinicalBERT alongside side the models that did not include pre-training on any clinical text.

Longformer is another modification based on BERT, the main appeal being the ability to take in longer sequences compared to BERT, resulting in fewer splits needed when the original text is long. Longformer also comes with a base- and large- format as BERT does. For this study, we adopted the large format (i.e. Longformer-large) which utilizes the same dimensionality per token as BERT-large to examine the effect of ingesting longer sequences and potentially improve performance.

### Information flow and implementation of the classification models

To set up the inputs for each model, the note sets (which can be long) are first split into “chunks,” whose length equals the maximum length each model could intake, with 20% overlapping tokens between a given chunk and its following chunk. Each note chunk inherits the label from its source note set. Two kinds of model outputs were extracted for each note chunk: (1) the modeled probability of treatment response corresponding to the input note chunk (provided by the language model in question, e.g., BERT, Longformer) belonging to the “improved” class, which can be directly converted to a modeled binary label for the chunk, and (2) an “embedding” of the note chunk (the output “[CLS] token” [9]), which is an array of decimal numbers containing information representing the note chunk (related to antidepressant treatment response).

For the sliding window approach, labels for the modeled note sets were determined through majority voting of their constituent note chunk labels. These chunk labels were derived by applying a fixed probability threshold of 0.5 to each chunk’s modeled probability. For the “hierarchical Transformer” approach, a second level light-weight Transformer model (eFigure 1) took in the embeddings of the note chunks produced by the first level model as its input, and outputted modeled note set labels. Each of the models was trained (or “fine-tuned” [9] because some models are pre-trained), and hyperparameters were optimized to maximize note set level accuracy (i.e. the proportion of agreement between the modeled and ground truth labels of the note set) on the training/validation sets. The modeling implementations were done using the Python programming language, and based on open-source, publicly available deep learning/NLP packages PyTorch [21] (for the implementation of Transformer encoders for the lightweight Transformer), Hugging Face Transformers [22], and SimpleTransformers [23] (for the implementation of the pre-trained BERT [9], ClinicalBERT [14], and Longformer [15]).

### Model training and evaluation

Models were trained (3296 note sets), tuned (350 note sets), and tested (350 note sets) using note sets exclusively from the main time window. For each model, we report the following performance on the hold-out test set of the main time window: AUROC and area under the precision-recall curve (AUPRC – PRC denotes the curve where the x-axis is sensitivity and the y-axis is positive predictive value (PPV)), both of which are threshold agnostic, as well as accuracy, PPV, negative predictive value (NPV), sensitivity, specificity, and F1 score (= 2 * (PPV * sensitivity)/(PPV + sensitivity)), based on a threshold chosen to maximize accuracy on the tuning (validation) set. Confidence intervals for the metrics were calculated based on bootstrapping the test set’s predicted probabilities and associated ground truth labels 500 times for each model. To evaluate model performance as a function of the total text available per patient, we report AUROC and AUPRC stratified by quartiles of note-set length (in tokens).

### Sensitivity analysis for model generalizability and robustness under distributional shifts

To assess model robustness to shifts in text distributions, we performed sensitivity analysis by evaluating the trained models on note sets from an additional “late time window” (12–26 weeks after the index date), capturing longer-term responses as treatment transitions into the maintenance phase.

In addition to differences in the timing of records relative to the index date—which could reflect distinct recording patterns associated with different post-treatment phases—the sampling for the late time window disproportionately included patients meeting the following criteria: participation in the MGB Biobank, initiation of antidepressant treatment after 1997, and having a primary care physician (PCP) within the MGB system. Previous research indicates that Biobank participants systematically differ from non-participants regarding healthcare utilization and clinical characteristics [24]. Additionally, patients with an internal PCP typically have more comprehensive medical documentation. Specifically, the proportions of patients meeting these criteria were approximately 2% in the main window (reflecting background prevalence), and 30% in the late window. The late time window, along with its corresponding sampling scheme and chart reviews, were initially designed and conducted as part of a separate study. Here, we leveraged these chart reviews to specifically evaluate model robustness to shifts in data distributions. A psychiatrist (YHS) reviewed note sets from a random sample of 376 eligible patients in the late time window. There was no overlap among patients across the two windows; in total, 4372 (3996 + 376) unique patients were included in the study. Model performance on these additional test sets was evaluated using the same metrics described previously.

## Results

Demographic characteristics of the sample are shown in Table 1. The chart review and data-splitting procedures resulted in the following distributions of positive labels across sets: 45% positive labels in the training set (main time window only), 46% in the validation set (main time window only), 45% for the main test set, and 52% for the sensitivity analysis (late time window) test set. These improvement proportions are consistent with real-world response estimates [25].

**Table 1:** Distribution of key demographics variables among for the patients constituting the note sets used for antidepressant treatment response phenotyping in the current study.

	All		Train		Tuning		Test		Late window	
Age	Mean	SD	Mean	SD	Mean	SD	Mean	SD	Mean	SD
	49.83	17.39	49.87	17.39	50.34	18.26	48.97	17.66	49.83	16.35
Gender	N	%	N	%	N	%	N	%	N	%
*Female*	2955	67.6%	2254	68.4%	250	71.4%	225	64.3%	226	60.1%
*Male*	1417	32.4%	1042	31.6%	100	28.6%	125	35.7%	150	39.9%
Language	N	%	N	%	N	%	N	%	N	%
*English*	3819	87.4%	2863	86.9%	300	85.7%	316	90.3%	340	90.4%
*Other*	466	10.7%	370	11.2%	42	12.0%	23	6.6%	31	8.2%
*Unknown*	87	2.0%	63	1.9%	8	2.3%	11	3.1%	5	1.3%
Marital status	N	%	N	%	N	%	N	%	N	%
*Married/Partner*	1877	43.0%	1417	43.0%	150	42.9%	151	43.1%	159	42.3%
*Separated/Divorced*	501	11.5%	368	11.2%	39	11.1%	40	11.4%	54	14.4%
*Single*	1543	35.3%	1159	35.2%	125	35.7%	129	36.9%	130	34.6%
*Widowed*	348	8.0%	273	8.3%	24	6.9%	23	6.6%	28	7.4%
*Other*	15	0.3%	14	0.4%	1	0.3%	0	0%	0	0%
*Unknown*	88	2.0%	65	2.0%	11	3.1%	7	2.0%	5	1.3%
Race	N	%	N	%	N	%	N	%	N	%
*African American*	290	6.6%	226	6.9%	24	6.9%	26	7.4%	14	3.7%
*Asian*	96	2.2%	73	2.2%	13	3.7%	8	2.3%	2	0.5%
*White*	3337	76.3%	2485	75.4%	256	73.1%	285	81.4%	311	82.7%
*Hispanic*	348	8.0%	272	8.3%	34	9.7%	14	4.0%	28	7.4%
*Other*	194	4.4%	162	4.9%	11	3.1%	9	2.6%	12	3.2%
*Unknown*	107	2.4%	78	2.4%	12	3.4%	8	2.3%	9	2.4%
Total N	4372		3296		350		350		376	

All: All patients included in the study. Train, Tuning, Test, Late Window: distribution stratified per data splits.

**Fig. 3:**
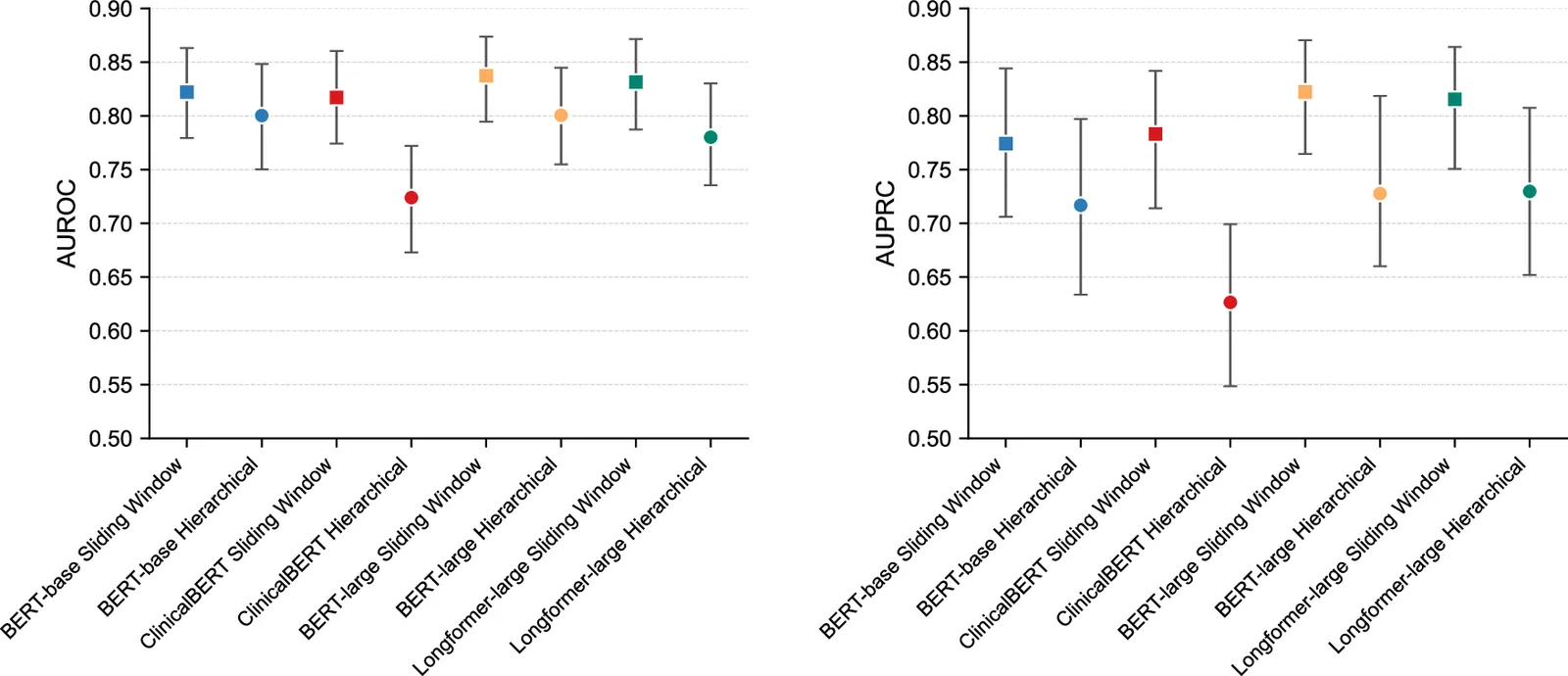
Point estimates and corresponding bootstrap confidence intervals for AUROC and AUPRC across modeling approaches. The left panel shows AUROC, and the right panel shows AUPRC. Squares indicate sliding-window models, and circles indicate hierarchical models. Colors denote model type: BERT-base, blue; ClinicalBERT, red; BERT-large, yellow; and Longformer, green. Error bars indicate confidence intervals.

Figure 3 shows point estimates and confidence intervals for AUROC and AUPRC on the main test set across the eight models (sliding-window or hierarchical Transformer variants using BERT-base, ClinicalBERT, BERT-large, and Longformer-large). Full numeric values for all classification performance metrics and their confidence intervals are provided in eTable 3. ROC curves for all eight models are provided in eFigure 2, and PRC curves are provided in eFigure 3. AUROC and AUPRC from the stratified analysis by token-length quartile are presented in eFigure 4.

The models performed well, with the majority of models achieving an AUROC greater than 0.80. For example, the BERT-large model with the sliding windows approach achieved an AUROC of 0.84, AUPRC of 0.82, PPV of 0.80, and NPV of 0.74 with a sensitivity of 0.63, specificity of 0.87, and F1 score of 0.70 at a decision threshold maximizing accuracy on the tuning (validation) set.

We also examined the impact of four key modeling decisions by comparing contrasting model configurations:

1. *Effect of pre-training the model on bio-clinical text (BERT-base vs ClinicalBERT):* ClinicalBERT shares the same model structure as BERT-base but is expected to contain more medical domain-specific information. However, as mentioned, this information was extracted from texts recorded in an ICU setting, which differs significantly from the majority of the sources for the notes included here. Overall, the additional fine-tuning on ICU notes during ClinicalBERT’s pre-training did not appear to materially change classification performance for antidepressant response in this task and dataset.
2. *Effect of larger input vector size per token and larger number of stacked Transformers (BERT-base vs BERT-large):* BERT-base and BERT-large share identical model architecture and pre-training methods but differ in the dimensions used per input token and the number of Transformer encoders stacked. Across configurations, BERT-large tended to show higher point estimates of performance than BERT-base, consistent with its larger representational capacity.
3. *Effect of larger maximum sequence length (BERT-large vs Longformer-large)*: BERT-large and Longformer-large performed similarly overall under both approaches, though BERT-large achieved slightly better performance based on point estimates. In hierarchical Transformer setups, both models concatenate two Transformer-based components; their primary difference relates to the interruption of information flow during training. Specifically, BERT-large processes a larger number of shorter text chunks compared to Longformer, resulting in more frequent arbitrary splits in the original text. However, this approach retains more total information as chunk representations enter the second-level Transformer, enabling richer interactions among chunks. When employing a sliding window approach, a model with more text splits (such as BERT-large) may lead to more granular classification decisions under a simple majority-voting mechanism.
4. *Examining the effect of sliding window vs hierarchical Transformer*: Models using the sliding-window approach generally showed higher point estimates of performance than hierarchical Transformer models. This pattern suggests that the additional complexity introduced by the second-level Transformer, compared to a simpler majority-voting mechanism, did not yield clear performance benefits for this task. Indeed, since the number of note chunks derived from each note set was relatively modest (eTable 4), complex models trained from scratch (such as the second-level Transformer models here) may not demonstrate clear advantages due to increased variance.

### Assessing the impact of token count

Based on point estimates, performance was generally stable across token-count strata, although there was a modest decrease in the highest token-count quantile (i.e., the longest note sets). Descriptively, this pattern appeared less pronounced for Longformer-based models, which may be related to their ability to accommodate longer input sequences per note chunk.

### Sensitivity analysis

Point estimates of classification performance metrics evaluated the late time window test set of each of the eight models are presented in eTable 5. Overall, despite minor decreases, the models maintained good performance, comparable to that seen using the main (4 – 12 week) time window (AUROCs ~0.75, with a maximum of 0.8 achieved by hierarchical Longformer-large), suggesting that model performance was broadly maintained in the presence of distributional shifts in the data. ROC curves for all eight models are provided in eFigure 5, and PRC curves are provided in eFigure 6.

## Discussion

We used data from a large healthcare system to evaluate deep learning-based NLP methods for classifying antidepressant response, a complex phenotype that relies on context-dependent patient and clinician reports without laboratory indicators. We systematically compared eight deep learning NLP modeling approaches that differed in handling of textual inputs. Despite the sparse nature of relevant information captured in clinical notes, these methods performed well overall, generally achieving AUROCs greater than 0.80. Models with larger token representations tended to show higher point estimates of performance. For example, the BERT-large model using a sliding window strategy achieved an AUROC of 0.84, a PPV of 0.80 at a specificity of 0.87. That is, there is an 80% probability that a patient classified as improved with antidepressant treatment would in fact be improved.

Several aspects of these results are noteworthy. This study is the first, to our knowledge, to apply deep learning-based NLP to phenotype antidepressant response in EHRs, and demonstrates that this approach can achieve strong classification performance. Conventionally, EHR-based phenotyping has relied on the following methods: 1) human expert review, which is not readily scalable; 2) term-based NLP, effectively an automated word search [8]; and/or 3) use of surrogate information e.g. ICD codes or other structured EHR data, which may not capture the complex and nuanced components of antidepressant response [26–28]. In contrast, the approach described here is highly scalable, more flexible than term-based NLP (by not requiring pre-specified terms), and uses clinically relevant information embedded in narrative notes, which are not directly encoded in structured EHR fields. Compared to term-based NLP or structured data alone, the current approach more closely emulates manual chart review – i.e., an artificial agent “reads through” the notes analogously to a human expert reviewer, taking in the same body of information. Because the information source is identical, this approach shares the same theoretical upper bound as expert review for outcome ascertainment, assuming sufficiently accurate modeling [9, 10, 13].

In addition to phenotyping treatment response, this “emulation” approach could facilitate automated EHR phenotyping of a broad range of conditions and may be particularly useful for psychiatric applications, where relevant information is often primarily recorded in free text using variable or inconsistent terms. In particular, key information may be incorporated in narratives about symptom change, documented clinician-patient discussions, descriptions of life events, clinician assessment and interpretations, all of which can be extracted through the modeling approach described here.

Our results should be interpreted with several limitations. First, chart review was conducted by a single psychiatrist because of the large volume of charts. Therefore, we could not estimate interrater reliability of the labeling process, that is, how consistently multiple reviewers would classify the same records, nor could we assess whether multi-rater adjudication would yield more stable reference labels or alter model performance. In the single-reviewer setting, disagreements between model-derived and manually assigned labels may reflect variation in reviewer judgment in addition to incorrect model classification of documented evidence of improvement. Nevertheless, even within this framework, the present models may already be useful for selected workflows tolerating and accounting for label noise, such as semi-supervised learning with smaller expert-adjudicated datasets or first-pass labeling in large-scale phenotyping studies. For example, model-estimated classification probabilities could support a triage workflow in which cases with high classification confidence are assigned model-derived labels, whereas cases with sub-threshold confidence are routed to manual review. Investigators could also manually review a stratified sample of high-confidence labeled cases to estimate misclassification within the automated-label strata, then combine these estimates with results from the manually reviewed sub-threshold cases to estimate an overall error rate for the hybrid labeling procedure. Whether model-derived labels are sufficiently accurate depends on the intended use case, selected operating threshold, consequences of outcome misclassification, and whether labels are used as final outcomes, proxy labels, or a triage mechanism. More error-sensitive applications, such as outcome ascertainment for emulated target trials, genetic discovery, or validation of clinical decision-support tools, would require further model refinement and benchmarking against multi-rater adjudicated reference standards when available. Second, like any supervised learning approach, our method still requires some manual chart review to derive training labels. Third, our models have not yet been directly tested on external datasets, so evaluating generalizability across health systems remains an important next step. Notably, our method readily supports fine-tuning models on local data, which could facilitate adaptation to new clinical sites.

Fourth, due to limited statistical power, we did not explicitly assess performance heterogeneity across demographic subgroups (e.g., race/ethnicity, gender). Future work in larger datasets may allow these analyses which will be important for assessing and mitigating any evidence of algorithmic bias. Fifth, the binary categorization of treatment response could be more granular: (1) the category “improvement” encompassed slight improvement to full remission; (2) patients without documented evidence of antidepressant response and those with insufficient or ambiguous documentation were combined into a single category (“No evidence of improvement”); and (3) we prioritized the last documented status within the 4–12 week window, consistent with how response is often operationalized in retrospective studies. Although this binary classification framework simplifies modeling and enhances clinical interpretability, it may obscure important heterogeneity within these categories and does not capture temporal variability in symptom course. Importantly, “improvement” in this study is not equivalent to scale-defined or DSM-defined remission, and the absence of documented improvement should not be interpreted as confirmed non-response. Rather, the latter category includes both true non-responders and patients with insufficient documentation and may therefore underestimate the true rate of treatment response. Future studies could benefit from a multiclass approach that explicitly differentiates true non-response from insufficient documentation, distinguishes partial responders from those achieving full remission, and more granularly captures temporal information. Sixth, interpreting large Transformer-based language models like BERT remains inherently complex, as current interpretability methods are not yet standardized or fully reliable [29, 30]. To enhance the clinical applicability of these classification models, future research should prioritize developing and evaluating robust interpretation techniques that can clarify model decision-making processes and strengthen confidence in their outputs.

Finally, our models are inherently constrained by the information available in the EHR. While EHRs contain abundant clinical details, they also have inherent limitations, including incomplete records, evolving documentation practices, variability in data entry, and potential recording biases. One implication is that, despite our efforts to restrict the sample to incident antidepressant users, the first recorded antidepressant prescription does not always indicate the patient’s true treatment initiation, as patients may have previously received care transition outside of the healthcare system. However, incident/prevalent user status would only influence the model’s behavior if there were systematic differences in how treatment response is recorded between the two groups.

Relatedly, because our goal was to evaluate whether deep learning-based NLP models can emulate an expert reviewer’s chart-review assessment of antidepressant response, we focused primarily on clinical notes, which form the basis of chart review in EHR phenotyping. While most structured data do not directly assess antidepressant treatment response [8], better integration of such data, including standardized questionnaires, could further enhance algorithm performance and provide useful information for model training and validation. Notably, although questionnaires appeared in a small proportion of patient notes (see Methods), they were converted into plain text without clear demarcations, obscuring their original tabular structure. This made questionnaire content difficult to extract and use reliably, either as structured model input or as an independent validation measure. Accordingly, it remains unclear whether questionnaire content meaningfully contributed to model performance here or merely introduced noise.

In summary, we applied deep learning-based approaches to phenotype antidepressant treatment response using real-world clinical notes and demonstrate that, despite challenges from sparse information and noisy formats, these approaches achieve strong classification performance. With further refinement, e.g., adopting larger language models with more parameters and pre-trained with more data, this method could enable high-throughput, semi-automated treatment phenotyping that would unlock a range of research and clinical applications.

## Data availabilty

Protected Health Information restrictions apply to the availability of the clinical data here, which were used under IRB approval for use only in the current study. As a result, this dataset is not publicly available. Qualified researchers affiliated with the Mass General Brigham (MGB) may apply for access to these data from the MGB EHR data repository (RPDR) through the MGB Institutional Review Board.

## Code availabilty

The pre-trained language model weights (BERT, ClinicalBERT, and Longformer), as well as their fine-tuning, were implemented directly using models and training procedures from the open-source Hugging Face Transformers [22] (4.2.2) and SimpleTransformers [23] (0.60.4) repositories without modifications. The second-level hierarchical Transformer was implemented using the Transformer encoder as provided by the PyTorch library (1.7.1) [21]. The source code of these packages and the pre-trained models is publicly available in their respective repositories.

## Supplementary information

Supplementary tables and figures

## References

[1] Hasin DS, Sarvet AL, Meyers JL, Saha TD, Ruan WJ, Stohl M, et al. Epidemiology of adult DSM-5 major depressive disorder and its specifiers in the United States. JAMA Psychiatry. 2018;75:336–46. 10.1001/jamapsychiatry.2017.4602 PMC5875313 29450462

[2] Dennis JM, Young KG, McGovern AP, Mateen BA, Vollmer SJ, Simpson MD, et al. Development of a treatment selection algorithm for SGLT2 and DPP-4 inhibitor therapies in people with type 2 diabetes: a retrospective cohort study. Lancet Digit Health. 2022;4:e873–83. 10.1016/S2589-7500(22)00174-1 36427949

[3] Dickerman BA, Gerlovin H, Madenci AL, Kurgansky KE, Ferolito BR, Figueroa Muñiz MJ, et al. Comparative effectiveness of BNT162b2 and mRNA-1273 Vaccines in U.S. Veterans. N Engl J Med. 2022;386:105–15. 10.1056/NEJMoa2115463 PMC8693691 34942066

[4] Xie Y, Bowe B, Al-Aly Z. Nirmatrelvir and risk of hospital admission or death in adults with covid-19: emulation of a randomized target trial using electronic health records. BMJ. 2023;381:e073312. 10.1136/bmj-2022-073312 PMC10086514 37041016

[5] Backman JD, Li AH, Marcketta A, Sun D, Mbatchou J, Kessler MD, et al. Exome sequencing and analysis of 454,787 UK Biobank participants. Nature. 2021;599:628–34. 10.1038/s41586-021-04103-z PMC8596853 34662886

[6] Verma A, Huffman JE, Rodriguez A, Conery M, Liu M, Ho YL, et al. Diversity and scale: genetic architecture of 2068 traits in the VA Million Veteran Program. Science. 2024;385:eadj1182. 10.1126/science.adj1182 PMC12857194 39024449

[7] Mehltretter J, Rollins C, Benrimoh D, Fratila R, Perlman K, Israel S, et al. Analysis of features selected by a deep learning model for differential treatment selection in depression. Front Artif Intell. 2019;2:31. 10.3389/frai.2019.00031 PMC7861264 33733120

[8] Perlis RH, Iosifescu DV, Castro VM, Murphy SN, Gainer VS, Minnier J, et al. Using electronic medical records to enable large-scale studies in psychiatry: treatment resistant depression as a model. Psychol Med. 2012;42:41–50. 10.1017/S0033291711000997 PMC3837420 21682950

[9] Devlin J, Chang M-W, Lee K, Toutanova K BERT: pre-training of deep bidirectional transformers for language understanding. In: Proceedings of the 2019 Conference of the North American Chapter of the Association for Computational Linguistics: Human Language Technologies, Volume 1 (Long and Short Papers). Minneapolis, Minnesota: Association for Computational Linguistics; 2019. pp. 4171–86. pp.

[10] Brown T, Mann B, Ryder N, Subbiah M, Kaplan J, Dhariwal P, et al. Language models are few-shot learners. Adv Neural Inf Process Syst. 2020;159:1877–901.

[11] Bushnell GA, Stürmer T, Mack C, Pate V, Miller M. Who diagnosed and prescribed what? Using provider details to inform observational research. Pharmacoepidemiol Drug Saf. 2018;27:1422–6. 10.1002/pds.4685 PMC6407693 30379369

[12] MassGeneralBrigham Healthcare System. Research Patient Data Registry (RPDR). https://rc.partners.org/about/who-we-are-risc/research-patient-data-registry. Accessed 1 Aug2018.

[13] Vaswani A, Shazeer N, Parmar N, Uszkoreit J, Jones L, Gomez AN et al. Attention is all you need. In: Proceedings of the 31st International Conference on Neural Information Processing Systems. NY, USA: Curran Associates Inc.: Red Hook; 2017. pp. 6000–10. pp.

[14] Alsentzer E, Murphy J, Boag W, Weng WH, Jin D, Naumann T et al. Publicly available clinical embeddings. In: Proceedings of the 2nd Clinical Natural Language Processing Workshop. Stroudsburg, PA, USA: Association for Computational Linguistics; 2019. pp. 72–78. pp.

[15] Beltagy I, Peters ME, Cohan A. Longformer: the long-document transformer. arXiv:200405150 [Preprint]. 2020. Availabl from: https://arxiv.org/abs/2004.05150.

[16] Pappagari R, Zelasko P, Villalba J, Carmiel Y, Dehak N. Hierarchical transformers for long document classification. In: 2019 IEEE Automatic Speech Recognition and Understanding Workshop (ASRU). Sentosa, Singapore, IEEE; 2019. p. 838–44.

[17] Goodfellow I, Bengio Y, Courville A. Deep learning. Cambridge, Massachusetts; The MIT Press: 2016.

[18] Davies M. Google Books Corpus. 2011. http://www.english-corpora.org/googlebooks/. Accessed 7 May2020.

[19] Davies M. The Wikipedia Corpus. 2015. https://www.english-corpora.org/wiki/. Accessed 5 May2020.

[20] Johnson AE, Pollard TJ, Shen L, Lehman LW, Feng M, Ghassemi M, et al. MIMIC-III, a freely accessible critical care database. Sci Data. 2016;3:160035. 10.1038/sdata.2016.35 PMC4878278 27219127

[21] Paszke A, Gross S, Massa F, Lerer A, Bradbury J, Chanan G, et al. PyTorch: an imperative style, high-performance deep learning library. In: Wallach H, Larochelle H, Beygelzimer A, dAlché-Buc F, Fox E, Garnett R (eds). Advances in Neural Information Processing Systems. Vancouver, Canada, Curran Associates, Inc.; 2019.

[22] Wolf T, Debut L, Sanh V, Chaumond J, Delangue C, Moi A, et al. Transformers: State-of-the-art natural language processing. In: Proceedings of the 2020 Conference on Empirical Methods in Natural Language Processing: System Demonstrations. Stroudsburg, PA, USA, Association for Computational Linguistics; 2020, p. 38–45.

[23] Rajapakse TC, Yates A, de Rijke M Simple transformers: open-source for All. In: Proceedings of the 2024 Annual International ACM SIGIR Conference on Research and Development in Information Retrieval in the Asia Pacific Region. NY, USA: ACM: New York; 2024. pp. 209–15. pp.

[24] Lee YH, Thaweethai T, Sheu Y-H, Feng YCA, Karlson EW, Ge T, et al. Impact of selection bias on polygenic risk score estimates in healthcare settings. Psychol Med. 2023;53:1–11. 10.1017/S0033291723001186 37226828

[25] Trivedi MH, Rush AJ, Wisniewski SR, Nierenberg AA, Warden D, Ritz L, et al. Evaluation of outcomes with citalopram for depression using measurement-based care in STAR*D: implications for clinical practice. Am J Psychiatry. 2006;163:28–40. 10.1176/appi.ajp.163.1.28 16390886

[26] Yu S, Chakrabortty A, Liao KP, Cai T, Ananthakrishnan AN, Gainer VS, et al. Surrogate-assisted feature extraction for high-throughput phenotyping. J Am Med Inf Assoc. 2017;24:e143–9. 10.1093/jamia/ocw135 PMC6080726 27632993

[27] Yu S, Ma Y, Gronsbell J, Cai T, Ananthakrishnan AN, Gainer VS, et al. Enabling phenotypic big data with PheNorm. J Am Med Inf Assoc. 2018;25:54–60. 10.1093/jamia/ocx111 PMC6251688 29126253

[28] Zhang Y, Cai T, Yu S, Cho K, Hong C, Sun J, et al. High-throughput phenotyping with electronic medical record data using a common semi-supervised approach (PheCAP). Nat Protoc. 2019;14:3426–44. 10.1038/s41596-019-0227-6 PMC7323894 31748751

[29] Lopardo G, Precioso F, Garreau D. Attention meets post-hoc interpretability: a mathematical perspective. In: Proceedings of the 41st International Conference on Machine Learning. Vienna, Austria, JMLR.org; 2024.

[30] Marjanovic S, Augenstein I, Lioma C Investigating the impact of model instability on explanations and uncertainty. In: Findings of the Association for Computational Linguistics ACL 2024. Stroudsburg, PA, USA: Association for Computational Linguistics; 2024. pp. 11854–79. pp.

