# Supplementary material for: Phenotyping antidepressant treatment response with deep learning in electronic health records
Source: Transl Psychiatry. 2026 Jul 11;16:525. doi: 10.1038/s41398-026-04266-1 (PMC13642340; doi:10.1038/s41398-026-04266-1)
Supplement: Supplementary file 1 [file 41398_2026_4266_MOESM1_ESM.docx]

**Supplemental Materials**

**eTable 1.** Glossary of terms defined, or related to deep learning appeared in this work

**eTable 2.** Percentiles of lengths per note set by tokens

**eTable 3.** Model performance result metrics with 95% bootstrapping confidence interval

**eTable 4.** Percentiles of chunks per note set

**eTable 5.** Classification performance metrics for the sensitivity analysis (late time window test set)

**eFigure legends**

**eFigure 1.** Model architecture of the “lightweight” Transformer applied in the hierarchical Transformer approach

**eFigure 2.** ROC plots for the eight models for the main test set

**eFigure 3.** Precision-recall (PR) plots for the eight models for the main test set

**eFigure 4.** AUROC and AUPRC for the main test set by token-length quartile

**eFigure 5.** ROC plots for the eight models for sensitivity analysis (late time window test set)

**eFigure 6.** Precision-recall (PR) plots for the eight models for sensitivity analysis (late time window test set)

**Supplemental References**

**eTable 1.** Glossary of terms defined, or related to deep learning appeared in this work

| **Term** | **Explanation** |
| --- | --- |
| *BERT*[^1^](https://sciwheel.com/work/citation?ids=11331484&pre=&suf=&sa=0&dbf=0) | BERT is a deep learning-based NLP model[^2^](https://sciwheel.com/work/citation?ids=11331712&pre=&suf=&sa=0&dbf=0) which is based on a sequence (or “stack”) of Transformers (defined below), heavily utilizing the self-attention mechanism (below). BERT is pre-trained on large amounts of general text such as Wikipedia, from which it learns general semantic and syntactical information before being applied for downstream applications. BERT comes in two different sizes, BERT-base and BERT-large, differing in the number of dimensions per text sequence (or “token”, below), and the total number of Transformers contained within their architectures. |
| *CLS token*[^1^](https://sciwheel.com/work/citation?ids=11331484&pre=&suf=&sa=0&dbf=0) | The CLS (standing for “classification”) token is a specific token built into both BERT and Longformer which summarizes information from all other individual tokens in the text sequence. |
| *ClinicalBERT*[^3^](https://sciwheel.com/work/citation?ids=7530095&pre=&suf=&sa=0&dbf=0) | ClinicalBERT is an extension of BERT-base which is further pre-trained on bio-medical text corpora, including MIMIC-III,[^4^](https://sciwheel.com/work/citation?ids=2590637&pre=&suf=&sa=0&dbf=0) an open source repository of clinical notes from the intensive care unit of an academic medical center. |
| *Deep learning*[^5^](https://sciwheel.com/work/citation?ids=11145762&pre=&suf=&sa=0&dbf=0) | Sometimes used interchangeably with deep neural networks (DNNs), deep learning is a modelling technique which takes inspiration from the emulation of biological neural systems. The foundational unit is the “artificial neuron” which is usually formed in a layer-wise structure – neurons in the same layer do not connect to each other, but would typically connect to the neurons in the adjacent layers. DNNs can approximate any arbitrary real function by sequentially combining (i.e., “stacking”) nonlinear layers[^6^](https://sciwheel.com/work/citation?ids=11331697&pre=&suf=&sa=0&dbf=0), and this flexibility is one source of their advantage |
| *Embedding* | A numerical vector that represents an entity by carrying some information about that entity; similar entities are represented by vectors that are numerically similar. The information used to represent the entity can vary in different contexts. In natural language processing, a basic form of information used to construct embeddings is co-occurrence of words, i.e., how frequently one word shows up adjacent to some other words. |
| *Feedforward layer*[^5^](https://sciwheel.com/work/citation?ids=11145762&pre=&suf=&sa=0&dbf=0) | A basic type of DNN within which every neuron in one layer is connected to every neuron in its previous and its next layer. |
| *Fine-tune*[^1^](https://sciwheel.com/work/citation?ids=11331484&pre=&suf=&sa=0&dbf=0) | The process of further training a pre-trained model (see below). Usually, the additional training is directly related to the task of interest and is less computationally intensive compared to the pre-training phase since the pre-trained model already contains relevant information (in NLP, the basic grammatical or syntactical information). |
| *Hierarchical Transformers*[^7^](https://sciwheel.com/work/citation?ids=11135617&pre=&suf=&sa=0&dbf=0) | A model architecture where the outputs of a first Transformer based model are used as the input of a second Transformer based model. The training of these two models is done in sequential steps. |
| *Hyperparameter* | The set of model characteristics that are adjustable rather than learned during training, such as the number of layers of the model. The process of optimizing hyperparameters by checking model performance on the validation set under different hyperparameter settings is termed “hyperparameter tuning.” |

**eTable 1.** Glossary of terms defined, or related to deep learning appeared in this work (continued)

| **Term** | **Explanation** |
| --- | --- |
| *Longformer*[^8^](https://sciwheel.com/work/citation?ids=11146095&pre=&suf=&sa=0&dbf=0) | Longformer is a class of deep learning-based NLP models that is an extension of BERT. It utilizes a modified self-attention mechanism which allows inputs of longer sequence lengths. |
| *Natural Language Processing (NLP)* | The technique that enables computers to process and analyze human language. |
| *Note chunk* | In the current study, a note chunk is a subsection of a note set whose length equals to maximum length allowed by either BERT or Longformer. |
| *Note set* | In the current study, a note set is a collection of all clinical notes recorded within a specified range of dates relative to the index date (e.g., 4-12 weeks after the index date) for a patient. |
| *Pre-train*[^1^](https://sciwheel.com/work/citation?ids=11331484&pre=&suf=&sa=0&dbf=0) | Before a specific application, a DNN model can be trained on an unrelated data set with a different task to learn general and lower level features that could benefit the actual task of interest. This process is called model pre-training (as opposed to “fine-tuning,” which is training the model further for the specific task of interest). For NLP, the model can be pre-trained to learn grammar and vocabulary meaning, etc., and thereby learn and carry that information before it is fine-tuned further for specific tasks. In most cases, the additional information learned from pre-training can be beneficial for a variety of downstream tasks, such as that demonstrated by BERT. |
| *Representation* | A transformation of input data (e.g., text) that carries information about the input data relevant to a particular task. An embedding (see above) is a type of representation in which elements of the input data are represented by a numerical vector. Similar representations imply similar content/meaning with respect to the task. |
| *Self-attention*[^9^](https://sciwheel.com/work/citation?ids=11145859&pre=&suf=&sa=0&dbf=0) | A process where representations of each token are expressed as a weighted sum of pair-wise similarities to every other token. Through this process, tokens that are relevant to the task are amplified (i.e., more strongly weighted) in the model predictions. |
| *Sliding window*[^7^](https://sciwheel.com/work/citation?ids=11135617&pre=&suf=&sa=0&dbf=0) | In the current study, sliding window denotes an approach to processing note sets that exceed the maximum length allowed by the model. These note sets are then divided into “chunks”, and labels (here, “improved” vs. “no evidence of improvement’) are assigned to each chunk. The final label for the overall note set is then given as the label that was more common across the chunks. |
| *Token*[^1^](https://sciwheel.com/work/citation?ids=11331484&pre=&suf=&sa=0&dbf=0) | A short sequence of characters that may be a number, a word, a fragment of a word, or a punctuation symbol that will then be converted into a vector to serve as an input to a model. The specific method of generating tokens from a given input text depends on the specific tokenizer applied. |

**eTable 1.** Glossary of terms defined, or related to deep learning appeared in this work (continued)

| **Term** | **Explanation** |
| --- | --- |
| *Tokenizer*[^1^](https://sciwheel.com/work/citation?ids=11331484&pre=&suf=&sa=0&dbf=0) | An algorithm which converts a text sequence into an array of tokens. In deep learning-based NLP, models are usually paired with a corresponding tokenizer. |
| *Transformer*[^9^](https://sciwheel.com/work/citation?ids=11145859&pre=&suf=&sa=0&dbf=0) | A model architecture originally developed for NLP which heavily utilizes the self-attention. It became the foundation of most contemporary deep learning- based NLP models, such as BERT, shortly after its invention. The essence of the Transformer architecture is a stack of alternating self-attention and feedforward layers. |

**eTable 2.** Percentiles of lengths per note set by tokens. The BERT family (BERT-base, BERT-large, and ClinicalBERT) shares a common way (“tokenizer”) of building tokens and therefore have the same number of tokens per note. Longformer uses a different tokenizer to build the tokens.

| Percentile | Token length per note set, BERT | Token length per note set, Longformer |
| --- | --- | --- |
| 1 | 199 | 171 |
| 10 | 597 | 521 |
| 25 | 1172 | 1015 |
| 50 | 3054 | 2634 |
| 75 | 8690 | 7420 |
| 90 | 20380 | 17649 |
| 99 | 71902 | 61580 |
|  |  |  |

**eTable 3.** Model performance result metrics with 95% bootstrapping confidence interval (main time window).

| **Model** | **Strategy** | **Accuracy** | **CI 95** |  | **AUROC** | **CI 95** |  | **AUPRC** | **CI 95** |  | **F1** | **CI 95** |  |
| --- | --- | --- | --- | --- | --- | --- | --- | --- | --- | --- | --- | --- | --- |
| BERT-base | sliding window | 0.73 | 0.69 | 0.78 | 0.82 | 0.78 | 0.86 | 0.77 | 0.71 | 0.84 | 0.72 | 0.66 | 0.77 |
| BERT-base | hierarchical | 0.73 | 0.68 | 0.77 | 0.80 | 0.75 | 0.85 | 0.72 | 0.63 | 0.80 | 0.67 | 0.60 | 0.73 |
| ClinicalBERT | sliding window | 0.73 | 0.68 | 0.77 | 0.82 | 0.77 | 0.86 | 0.78 | 0.71 | 0.84 | 0.72 | 0.67 | 0.77 |
| ClinicalBERT | hierarchical | 0.67 | 0.62 | 0.71 | 0.72 | 0.67 | 0.77 | 0.63 | 0.55 | 0.70 | 0.64 | 0.57 | 0.69 |
| BERT-large | sliding window | 0.76 | 0.72 | 0.81 | 0.84 | 0.79 | 0.87 | 0.82 | 0.76 | 0.87 | 0.70 | 0.65 | 0.76 |
| BERT-large | hierarchical | 0.76 | 0.72 | 0.81 | 0.80 | 0.75 | 0.84 | 0.73 | 0.66 | 0.82 | 0.74 | 0.68 | 0.79 |
| Longformer-large | sliding window | 0.77 | 0.72 | 0.80 | 0.83 | 0.79 | 0.87 | 0.82 | 0.75 | 0.86 | 0.72 | 0.66 | 0.77 |
| Longformer-large | hierarchical | 0.74 | 0.69 | 0.79 | 0.78 | 0.74 | 0.83 | 0.73 | 0.65 | 0.81 | 0.67 | 0.61 | 0.73 |
|  |  |  |  |  |  |  |  |  |  |  |  |  |  |
| **Model** | **Strategy** | **NPV** | **CI 95** |  | **PPV** | **CI 95** |  | **Sensitivity** | **CI 95** |  | **Specificity** | **CI 95** |  |
| BERT-base | sliding window | 0.79 | 0.73 | 0.84 | 0.68 | 0.61 | 0.74 | 0.76 | 0.69 | 0.83 | 0.71 | 0.65 | 0.77 |
| BERT-base | hierarchical | 0.73 | 0.66 | 0.79 | 0.73 | 0.65 | 0.81 | 0.62 | 0.54 | 0.70 | 0.81 | 0.75 | 0.86 |
| ClinicalBERT | sliding window | 0.80 | 0.73 | 0.86 | 0.67 | 0.59 | 0.73 | 0.79 | 0.72 | 0.85 | 0.68 | 0.61 | 0.74 |
| ClinicalBERT | hierarchical | 0.71 | 0.64 | 0.78 | 0.62 | 0.55 | 0.69 | 0.66 | 0.58 | 0.73 | 0.67 | 0.61 | 0.73 |
| BERT-large | sliding window | 0.74 | 0.68 | 0.80 | 0.80 | 0.72 | 0.86 | 0.63 | 0.56 | 0.71 | 0.87 | 0.82 | 0.92 |
| BERT-large | hierarchical | 0.79 | 0.73 | 0.85 | 0.73 | 0.66 | 0.81 | 0.75 | 0.67 | 0.81 | 0.78 | 0.71 | 0.84 |
| Longformer-large | sliding window | 0.76 | 0.70 | 0.82 | 0.77 | 0.70 | 0.84 | 0.68 | 0.59 | 0.75 | 0.84 | 0.78 | 0.89 |
| Longformer-large | hierarchical | 0.72 | 0.66 | 0.78 | 0.78 | 0.71 | 0.86 | 0.59 | 0.52 | 0.66 | 0.87 | 0.81 | 0.92 |

**eTable 4.** Percentiles of chunks per note set. The BERT family (BERT-base, BERT-large, and ClinicalBERT) shares a common way of building tokens. They also have a smaller limit of sequence length (512 tokens) and therefore resulted in more chunks per note set compared to Longformer-large (sequence length limit of 4,096 tokens).

| Percentile | Number of chunks per note set, BERT | Number of chunks per note set, Longformer |
| --- | --- | --- |
| 1 | 1 | 1 |
| 10 | 2 | 1 |
| 25 | 3 | 1 |
| 50 | 8 | 1 |
| 75 | 22 | 3 |
| 90 | 51 | 6 |
| 99 | 180 | 19 |
|  |  |  |

**eTable 5.** Classification performance metrics for each model on the hold-out test set from the late time window (n=376). For threshold-dependent metrics (accuracy, F1, NPV, PPV, sensitivity, specificity), for each model we chose the model probability threshold that maximized the accuracy of label binarization. Hierarchical: Hierarchical Transformers modeling approach.

| **Model** | **Strategy** |  | **Accuracy** | **AUROC** | **AUPRC** | **F1** | **NPV** | **PPV** | **Sensitivity** | **Specificity** |
| --- | --- | --- | --- | --- | --- | --- | --- | --- | --- | --- |
| BERT-base | *sliding window* |  | 0.67 | 0.72 | 0.72 | 0.70 | 0.68 | 0.67 | 0.73 | 0.62 |
|  | *hierarchical* |  | 0.67 | 0.72 | 0.71 | 0.65 | 0.63 | 0.73 | 0.59 | 0.76 |
| ClinicalBERT | *sliding window* |  | 0.67 | 0.69 | 0.70 | 0.69 | 0.67 | 0.67 | 0.70 | 0.64 |
|  | *hierarchical* |  | 0.63 | 0.66 | 0.65 | 0.63 | 0.61 | 0.65 | 0.62 | 0.64 |
| BERT-large | *sliding window* |  | 0.68 | 0.77 | 0.79 | 0.63 | 0.63 | 0.79 | 0.53 | 0.85 |
|  | *hierarchical* |  | 0.73 | 0.76 | 0.76 | 0.73 | 0.70 | 0.76 | 0.70 | 0.76 |
| Longformer-large | *sliding window* |  | 0.74 | 0.80 | 0.82 | 0.74 | 0.71 | 0.79 | 0.69 | 0.80 |
|  | *hierarchical* |  | 0.69 | 0.72 | 0.73 | 0.66 | 0.64 | 0.78 | 0.57 | 0.83 |

**eFigure legends**

**eFigure 1.** Model architecture of the “lightweight” Transformer applied in the hierarchical Transformer approach. Positional encoding: adding positional marker into the input sequence which enables the model to identify the position of each input token. Norm: normalization to range between 0 and 1. The Transformer architecture (gray box) was described in Vaswani et. al. 2017.[^9^](https://sciwheel.com/work/citation?ids=11145859&pre=&suf=&sa=0&dbf=0)

**eFigure 2.** ROC plots for the eight models for the main time window 1. e2.1.1 – BERT-base, sliding window; e2.1.2 – BERT-base, hierarchical Transformers; e2.2.1 – ClinicalBERT, sliding window; e2.2.2 – ClinicalBERT, hierarchical Transformers; e2.3.1 – BERT-large, sliding window; e2.3.2 – BERT-large, hierarchical Transformers; e2.4.1 – Longformer, sliding window; e2.4.2 – Longformer, hierarchical Transformers.

**eFigure 3.** Precision-recall (PR) plots for the eight models for the main time window. e3.1.1 – BERT-base, sliding window; e3.1.2 – BERT-base, hierarchical Transformers; e3.2.1 – ClinicalBERT, sliding window; e3.2.2 – ClinicalBERT, hierarchical Transformers; e3.3.1 – BERT-large, sliding window; e3.3.2 – BERT-large, hierarchical Transformers; e3.4.1 – Longformer, sliding window; e3.4.2 – Longformer, hierarchical Transformers. Color gradients indicate the decision threshold applied for label binarization, moving between a real value between 0 and 1.

**eFigure 4.** AUROC and AUPRC for the main test set by token-length quartile

**eFigure 5.** ROC plots for the eight models for the late time window. e5.1.1 – BERT-base, sliding window; e5.1.2 – BERT-base, hierarchical Transformers; e5.2.1 – ClinicalBERT, sliding window; e5.2.2 – ClinicalBERT, hierarchical Transformers; e5.3.1 – BERT-large, sliding window; e5.3.2 – BERT-large, hierarchical Transformers; e5.4.1 – Longformer, sliding window; e5.4.2 – Longformer, hierarchical Transformers.

**eFigure 6.** Precision-recall (PR) plots for the eight models for the late time window. E6.1.1 – BERT-base, sliding window; e6.1.2 – BERT-base, hierarchical Transformers; e6.2.1 – ClinicalBERT, sliding window; e6.2.2 – ClinicalBERT, hierarchical Transformers; e6.3.1 – BERT-large, sliding window; e6.3.2 – BERT-large, hierarchical Transformers; e6.4.1 – Longformer, sliding window; e6.4.2 – Longformer, hierarchical Transformers. Color gradients indicate the decision threshold applied for label binarization, moving between a real value between 0 and 1.

**eFigure 1.**

**eFigure 2.**

eFigure 2.1.1

eFigure 2.1.2

eFigure 2.2.1

eFigure 2.2.2

eFigure 2.3.1

eFigure 2.3.2

eFigure 2.4.1

eFigure 2.4.2

**eFigure 3.**

eFigure 3.1.1

eFigure 3.1.2

eFigure 3.2.1

eFigure 3.2.2

eFigure 3.3.1

eFigure 3.3.2

eFigure 3.4.1

eFigure.3.4.2

**eFigure 4.**

**eFigure 5.**

eFigure 5.1.1

eFigure 5.1.2

eFigure 5.2.1

eFigure 5.2.2

eFigure 5.3.1

eFigure 5.3.2

eFigure 5.4.1

eFigure 5.4.2

**eFigure 6.**

eFigure 6.1.1

eFigure 6.1.2

eFigure 6.2.1

eFigure 6.2.2

eFigure 6.3.1

eFigure 6.3.2

eFigure 6.4.1

eFigure 6.4.2

**Supplemental References**

[1.    Devlin, J., Chang, M.-W., Lee, K. & Toutanova, K. BERT: Pre-training of Deep Bidirectional Transformers for Language Understanding. in *Proceedings of the 2019 Conference of the North American Chapter of the Association for Computational Linguistics: Human Language Technologies, Volume 1 (Long and Short Papers)* 4171–4186 (Association for Computational Linguistics, 2019).](https://sciwheel.com/work/bibliography/11331484)

[2.    Socher, R., Bengio, Y. & Manning, C. D. Deep Learning for NLP (without Magic). in *Tutorial Abstracts of ACL 2012* 5 (Association for Computational Linguistics, 2012).](https://sciwheel.com/work/bibliography/11331712)

[3.    Alsentzer, E. *et al.* Publicly Available Clinical Embeddings. in *Proceedings of the 2nd Clinical Natural Language Processing Workshop* 72–78 (Association for Computational Linguistics, 2019). doi:10.18653/v1/W19-1909.](https://sciwheel.com/work/bibliography/7530095)

[4.    Johnson, A. E. W. *et al.* MIMIC-III, a freely accessible critical care database. *Sci. Data* **3**, 160035 (2016).](https://sciwheel.com/work/bibliography/2590637)

[5.    Goodfellow, I., Bengio, Y. & Courville, A. *Deep learning*. (The MIT Press, 2016).](https://sciwheel.com/work/bibliography/11145762)

[6.    Baker, M. R. & Patil, R. B. Universal Approximation Theorem for Interval Neural Networks. *Reliable Computing* (1998).](https://sciwheel.com/work/bibliography/11331697)

[7.    Pappagari, R., Zelasko, P., Villalba, J., Carmiel, Y. & Dehak, N. Hierarchical transformers for long document classification. in *2019 IEEE Automatic Speech Recognition and Understanding Workshop (ASRU)* 838–844 (IEEE, 2019). doi:10.1109/ASRU46091.2019.9003958.](https://sciwheel.com/work/bibliography/11135617)

[8.    Beltagy, I., Peters, M. E. & Cohan, A. Longformer: The Long-Document Transformer. *arXiv:2004.05150* (2020).](https://sciwheel.com/work/bibliography/11146095)

[9.    Vaswani, A. *et al.* Attention is All You Need. in *Proceedings of the 31st International Conference on Neural Information Processing Systems* 6000–6010 (Curran Associates Inc., 2017).](https://sciwheel.com/work/bibliography/11145859)
